# Awareness Programs and Change in Taste-Based Caste Prejudice

**DOI:** 10.1371/journal.pone.0118546

**Published:** 2015-04-22

**Authors:** Ritwik Banerjee, Nabanita Datta Gupta

**Affiliations:** Aarhus University, Aarhus, Denmark; Middlesex University London, UNITED KINGDOM

## Abstract

Becker's theory of taste-based discrimination predicts that relative employment of the discriminated social group will improve if there is a decrease in the level of prejudice for the marginally discriminating employer. In this paper we experimentally test this prediction offered by Garry Becker in his seminal work on taste based discrimination, in the context of caste in India, with management students (potential employers in the near future) as subjects. First, we measure caste prejudice and show that awareness through a TV social program reduces implicit prejudice against the lower caste and the reduction is sustained over time. Second, we find that the treatment reduces the prejudice levels of those in the left tail of the prejudice distribution - the group which can potentially affect real outcomes as predicted by the theory. And finally, a larger share of the treatment group subjects exhibit favorable opinion about reservation in jobs for the lower caste.

## Introduction

Caste is an age-old system of rigid social and ritual stratification of Indian society, implying the total exclusion of certain groups from the rights and opportunities for advancement. Discrimination based on caste continues to have very deep roots in India and thus presents a good setting for studying the origins of discrimination. At the same time, quota-based affirmative action policy has been adopted in a larger scale in India than elsewhere with reservation of public sector jobs, political seats and slots in higher educational institutions for the lowest-caste groups in society. No reservation however exists in the private sector. Despite a social setting different from that in which racial prejudice against African Americans is observed, the issue of caste-based discrimination can be studied under the framework of the Beckerian theory [1] of taste biases.

Becker’s theory makes an important prediction—a decrease in prejudice level against a social group will lead to an increase in employment and reduction in wage inequality for that social group but it is not the average prejudice level but the prejudice level held by the marginally discriminating employer, which matters. However the implication of this prediction has not been analyzed in the context of caste in India. Furthermore, few studies are able to exogenously manipulate the prevalent prejudice levels and thereby cleanly identify effect of prejudice on wage and hiring outcomes. This problem is further compounded by the fact that the menu of possible interventions by which prejudice may be affected is rather limited.

In this paper we experimentally test the predictions offered by Becker in the context of caste, with management students (potential employers in the near future) as subjects. We design a unique lab-based experiment by which prejudice levels of the subjects are exogenously altered. Our experiment exposes groups of observationally equivalent Masters level business school students at a prominent business school in Delhi to either a TV program on caste injustices or an innocuous TV cartoon program. Their prejudice levels are then measured by a caste relevant Implicit Attitude Test (IAT; [2]) available through the Project Implicit (a multi-university research collaboration started in 1998 by aimed at systematically developing methods to elicit and measure automatic preferences) website and their opinions regarding job reservation recorded.

Our results show that the treated group who watch the TV program show significantly lower implicit bias and state a significantly higher preference for private sector job reservation. These effects persist even 3 months after the treatment is carried out. We rule out that the changed behavior in our study is induced by a celebrity effect of a famous Bollywood movie star hosting the program. Instead, we show that the treatment shifts the prejudice distribution of our sample of highly-educated business school students (likely future employers) mainly at the lower tail. This fact, coupled with the additional finding that the treatment significantly increases a desire for private sector reservation indicates that caste-based discrimination in India by high-educated urban individuals is taste-based.

Thus our study is one of the first to characterize the nature of employer discrimination related to caste by exogenously manipulating prejudice levels in the lab. We also show that public awareness campaigns play an important instrumental role in reducing caste prejudice and its effect is persistent over time.

The rest of the paper is organized as follows: section 2 discusses the related literature, section 3 sketches a theoretical framework, section 4 lays out the experimental design and procedure, section 5 presents results, validation exercises and considers mechanisms and Section 6 provides a brief conclusion.

## Related Literature

An extensive literature on racial discrimination largely looking at the black-white differences in the US has evolved over the years (see [3]). Few empirical studies, however, directly measure taste-based prejudice and relate it labor market inequalities. [4] who study the black-white wage gap in the US find that one quarter of the racial wage gap can be explained by prejudice. [5] find similar results in the context of the ethnic wage gap in Sweden. In India, despite legislated policies on affirmative action, there still remains substantial employer discrimination by caste (documented by [6], [7]). But we know little about the nature of the employer discrimination and what strategies to pursue to ameliorate employment conditions of the low caste. Unlike most studies which rely on evidence for discrimination indirectly (e.g. [8] and [9] look at differences in the belief distribution and altruistic preference respectively), IAT allows us to directly measure levels of prejudice. [10] takes this idea to Swedish data and relates automatic preferences with discrimination in hiring immigrants in Sweden.

We also draw on another emerging literature showing that public awareness programs distributed via social media have a big impact on behavior. Such programs generally operate by changing the social norm as opposed to through purely economic channels such as price, income, quota etc. For instance in Brazil [11] finds that a television soap opera portraying small families lead to significantly lower fertility among women who are exposed to it. Such an effect has also been found by [12] who combine Nielsen ratings, Google searches and Twitter tweets following a US TV program (16 and Pregnant) documenting the late-term pregnancies and early days of motherhood of teen mothers. They quantify its effects on teen childbearing outcomes and attribute a 5.7% reduction in teen childbearing in the 18 months following the program which amounts to around one third of the overall decline in teen childbearing over this period. While this study found a longer lasting effect of a TV social program on behavior, other programs (paid political advertising via television ads) had short-lived effects on (voter) preferences such as the one analyzed by [13].

Psychologists have taken this a layer deeper by examining how such interventions affect our behavioral primitive—automatic preference, which in turn aggregates to social norms. Such automatic preference is unwitting, unintentional and uncontrollable and thereby different from explicit preference; yet it has real consequence in terms of affecting social judgment and real outcomes such as hiring decisions [10]. [2] found that diversity education administered by a likeable black professor over a period of 14 weeks reduced implicit prejudice by increasing contact with black students. Even in the short term, [14] find that situational exposure to positive gay role models reduced implicit prejudice against gay and lesbian people (For an excellent review of the literature on psychology see [15]). In fact one of the early studies in Economics to use automatic preferences in understanding discrimination, [16], notes that “manipulating” implicit preference and thereby remedying implicit discrimination is feasible and important as discriminating behavior can be partially reduced without requiring the discriminating agent to act against her will.

An IAT is a widely used computer based tool, designed to capture the automatic preferences vis-a-vis social groups, products or identity. It compares the speed of categorization in two different sorting conditions [17]. For example, the concepts “Low Caste” and “Bad” tend to be more strongly associated than the concepts “Low Caste” and “Good”; thus respondents are able to identify and categorize items faster in a condition in which items represent “Low Caste” and “Bad” than in which items representing “Low Caste” and “Good”. The test computes scores by calculating the difference in speed of association between the two sorting conditions. The details of the scoring algorithm can be found in [18].

In this paper we target a group of likely future Indian employers—the graduates of two prestigious business schools in Delhi—and explore their implicit attitudes relating to caste and test whether we can experimentally affect such attitudes and more directly, hiring preferences. Our aim is to look deeper into the nature of employer caste bias in India, its consequences on real labor market outcomes and instruments by which caste bias can be reduced.

## Theoretical Framework

In this section we revisit the seminal work of [1] on discrimination to examine what type of clear prediction can be derived in terms of employment and wage inequality in the presence of discrimination. Models of statistical discrimination (due to [19]), the alternative theory of discrimination, cannot generate observed patterns in employment and unemployment (for a review of how the two strands of theory compare see [20]). Thus taste-based discrimination models remain the most relevant tool to study hiring preferences of employers.

Clearly, the dynamics of caste discrimination may differ in certain respects from other kinds of ethnic discrimination and in-group favoritism (especially race which motivated Becker’s analysis). One major difference between caste and race is that while race is visually identified on the basis of ascriptive differences between groups (skin colour, facial features etc), caste is a non-ascriptive system [21, 22]. Caste on the other hand, is identified via last name which indicates social group affiliation in an Indian context, and thus readily known by prospective employers. Recent studies indicate that name-based discrimination also exists for race in the US as certain first names are more widely used by certain races [23]. This resume-based study showed that ‘white’ names received 50% more call-back than ‘black’ names and employers reacted simply to the names and not to the content of the resumes, which were otherwise identical. Even though there are certain differences between how race and caste is visually perceived, the point is that each system is associated with deep historical segregation, lack of economic opportunity and widespread discrimination. In both cases, governments have made large efforts to eradicate these persistent differences in treatment via affirmative action programs [22]. Since Becker’s model requires only that employers are willing to pay something to be able to associate with majority workers, the model applies equally well to caste discrimination as to race discrimination.

We assume that employers belong to the upper caste but workers may be hired from either low caste (*l*) or high caste (*h*). An employer’s utility is *V*_*i*_ and it depends on the profit earned *P*_*i*_ and the taste disutility *d*_*i*_ from employing a lower caste laborer (let us assume that *d*_*i*_ ∼ *F*(.) with support from 0 to 1). Thus *V*_*i*_ = *P*_*i*_−*d*_*i*_ *L*_*l*_ where *P*_*i*_ = *f*(*L*_*l*_+*L*_*h*_)−*w*_*l*_ *L*_*l*_−*w*_*h*_ *L*_*h*_ is the earned profit from employing *L*_*l*_ low caste and *L*_*h*_ upper caste labor and *f*(.)is a CRS production function. Employers maximize *V*_*i*_ by choosing *L*_*l*_ and *L*_*h*_. For interior solution $L_{l}^{*},L_{h}^{*}>0$ the first order conditions are $f^{\prime }(L_{l}^{*}+L_{h}^{*})=w_{l}+d_{i}$ and $f^{\prime }(L_{l}^{*}+L_{h}^{*})=w_{h}$. Thus an upper caste employer endowed with a taste disutility *d*_*i*_, faces a perceived marginal cost of employing a low caste laborer *w*_*l*_+*d*_*i*_, whereas he faces *w*_*h*_ when facing a high caste laborer. Thus *i* hires a high caste laborer as long as *w*_*h*_ < *w*_*l*_+*d*_*i*_ and a low caste laborer otherwise. Market clearing condition implies that $w_{h}^{*}$ and $w_{l}^{*}$ is obtained through equating labor supply and demand and the employer who is an indifferent discriminator, indexed by d*, is the one for whom $w_{h}^{*}=w_{l}^{*}+d^{*}$. Thus all employers with *d*_*i*_ > d* i.e. of mass 1−*F*(d*) will employ upper caste workers and those with *d*_*i*_ < d* i.e. of mass *F*(d*) will employ lower caste. Becker terms the employer prejudice level d* as the “marginally discriminating employer”.

Now let us consider a thought experiment where due to some external intervention the distribution of *d*_*i*_ shifts to the left i.e. *d*_*i*_ ∼ *G*(.) such that *G*(*d*_*i*_) > *F*(*d*_*i*_), ∀*i*.

Prediction 1: The proportion of employers who will be willing to hire lower caste labor will be greater under *G*(.) than under *F*(.) i.e. *G*(d*) > *F*(d*).

Prediction 2: The wage inequality between lower and higher caste labor will be lower (i.e. *w*_*l*_/*w*_*h*_ will be higher) under *G*(.) than under *F*(.).

Prediction 3: The extent of labor market inequalities (wage and employment) is driven by the discrimination level of the marginally discriminating employer.

Prediction 3 offers the important insight that wage and employment gaps should be systematically related not to the average prejudice level but to the marginal discriminator’s prejudice level. It is difficult to empirically validate this prediction as it is hard to obtain the distribution of prevailing prejudice, which is further compounded by the fact that prejudice itself is difficult to measure precisely. It is even harder to know who the marginal discriminator is. Becker provides a simple rule of thumb that under some (not-so-outrageous) conditions, the marginal discriminator is the one who has the *p-th* percentile prejudice level given that the *p* is the proportion of the discriminated group in the population. In other words, if the share of the lower caste in India’s population is 25% then the marginally discriminating upper caste employer is the one who has the 25th percentile prejudice level.

Closely tied to this theory, our study addresses three important questions. First, our experiment is one of the first to measure the (implicit) prejudice distribution among potential employers in the private corporate sector. Second, we aim to understand whether the instrument of awareness program is an effective policy instrument in so far as reducing the distribution of prejudice among potential employers. Third and most importantly, we examine which percentile the change in the prejudice distribution, if any, is coming from—this allows us to verify if the designed intervention is able to reduce the prejudice levels of the marginally discriminating employer. Getting more lower caste labor hired by high caste employers is possible only when the change in distribution of *d*_*i*_ is driven by a change in the marginally discriminating employer and not the average level of discrimination held by all employers. Finally, we cannot observe actual hiring decisions of the student subjects but we can proxy it by their opinion about job reservation. While admittedly the two are not the same, opinion about job reservation can be a good indicator of a future manager’s preference about a low caste person’s inclusion in the labor force. With that caveat, we shall analyze Prediction 1 i.e. if a shift in the distribution of *d*_*i*_ affects opinion about reservation-based affirmative action.

## Experiment Design and Procedure

Subjects are randomized into two treatment groups, which differ in the content of the video they watch. In one treatment they watch parts of an episode of a reality TV show—Satya Meva Jayate (SMJ), which documents the atrocities often meted out to lower caste people in India and the inequality of opportunity that follows. It further attempts to deliver a strong social message which indicts caste-based discriminatory behavior.

SMJ hosted by one of the most popular film stars in Bombay, was widely watched across India and dealt with a socially pressing issue in each episode. “Dignity for All” was one of the episodes and it dealt with caste-based social segmentation and discrimination in India. Subjects watch the most relevant 30 minutes of the one hour long episode “Dignity for All” from the website of Satya Meva Jayate. The show was divided into three broad segments, interspersed with strong nudges towards an ideal moral behavior. In segment one, people from the low castes were invited to narrate their experiences of being Dalit or low caste persons. Besides sharing the inhuman treatments meted out to them or their family owing to their lower caste, they also shared their views on the current state of affairs and the future course. The experiences were narrated in emotionally charged language, also known as “loaded language” in the experimental literature. [24] for instance studies the effect of framing in a different context. The emotionally charged nature of narrations was an important element in reduction in prejudice levels. In the second segment a former Justice of Supreme Court, also a Gandhian, was invited. He enunciated that the ideas of equality, fraternity, liberty and justice were enshrined in India’s Constitution, but also reflected on how these ideas were being violated. In the third segment, a documentary filmmaker shared his personal anecdotal experiences of working in eight different states, besides some statistical figures, and confirmed wide prevalence of deeply discriminatory tendencies.

In the control group subjects watch an episode of Tom and Jerry for thirty minutes. Tom and Jerry is chosen as a counterfactual as it has little cultural moorings and thus is likely to provide subjects with a neutral reference frame. The video series with universal appeal, consists of an ongoing cat and mouse chase, where the less clever but stronger and larger cat tries in vain to capture the tiny yet clever mouse. It turns out that more often than not, it is the tiny mouse which outwits the cat in the series. Thus not only the *cartoonisque* manner of representation but also the the characters exhibit no hierarchical content and thus is an appropriate counterfactual for us. If anything, it is a show of solidarity with the weak (mouse) and therefore may only bias the IAT ranks upward and thereby negate the treatment effect.

**Fig 1:**
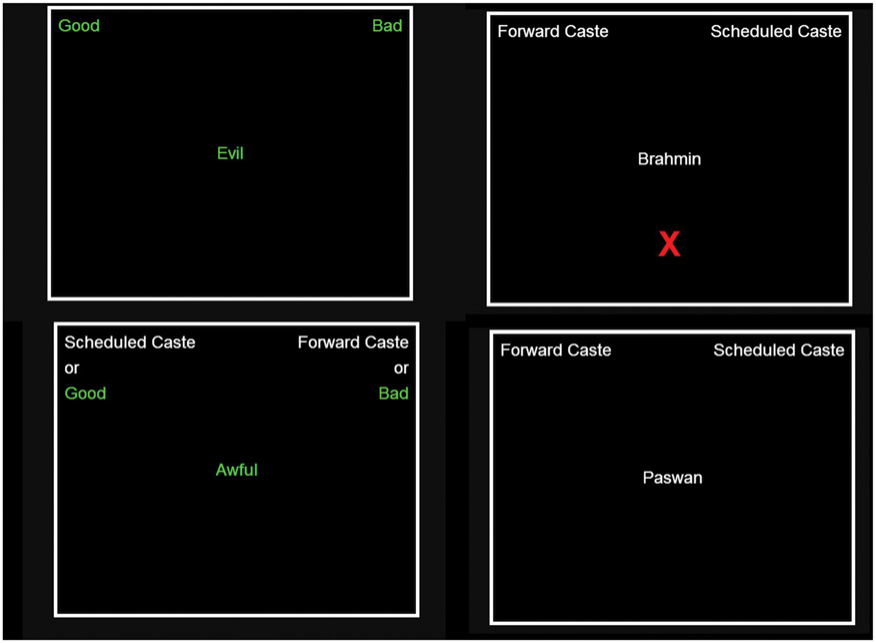
Screen shots from IAT. The picture shows four different screenshots from the IAT. Subjects were required to associate the words that came up with either the left or the right category.

**Fig 2:**
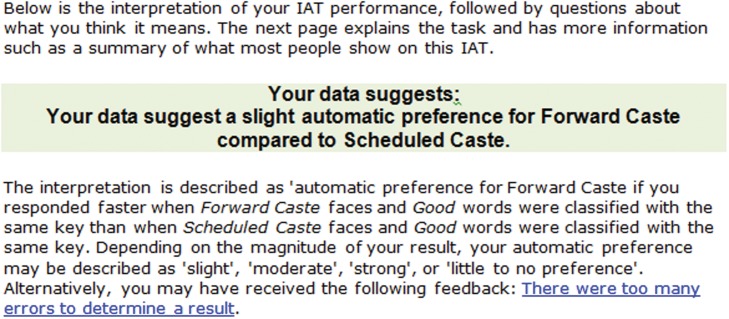
Result screen shot. The picture is a screen shot example of the result.

After watching the video for thirty minutes, subjects are directed to the Project Implicit website where they are asked to take the Caste—IAT (see Fig 1 for screenshots of the test). It is specifically designed to capture caste-based automatic preferences. In the test, subjects quickly categorize different word based stimuli (in our case it is surnames which denote caste) into left or right categories. If the stimulus belongs to the right (left) category, the right (left) key has to be pressed. An implicit prejudice against low caste will potentially show up as a response time differential. Based on this, IAT scores are computed and results generated. We ask the students to write down the results on the response sheet. It is important to note that the test scores are not observed, but what one observes is only the result of the test-taker. It is important to note that the results from the test are stated as one of the seven alternatives. Since a subject sees one and only one of the following alternatives (see the Appendix for a screen-shot), it is impossible for her to falsify the true result and write something socially more desirable. In order to further prevent any falsification, the experimenter randomly matched the result on the computer screen with the one that the subject writes down on paper. The result (see Fig 2 for a screenshot) is stated as one of the following seven alternatives -

- Strong automatic preference for Scheduled Caste compared to Forward Caste (level = 3)
- Moderate automatic preference for Scheduled Caste compared to Forward Caste (level = 2)
- Slight automatic preference for Scheduled Caste compared to Forward Caste (level = 1)
- No preference of for one caste over another (level = 0)
- Slight automatic preference for Forward Caste compared to Scheduled Caste (level = -1)
- Moderate automatic preference for Forward Caste compared to Scheduled Caste (level = -2)
- Strong automatic preference for Forward Caste compared to Scheduled Caste (level = -3)

For the purpose of empirical analysis, we convert the result into numerical scores levels as indicated above. The above design allows us to draw treatment effects between results from caste-based IAT score of those who watch the episode “Dignity for All” and that of those who watch Tom and Jerry.

In order to address whether the treatment effect is sustained over time, we followed the participants in the treatment over time. They take the caste-based IAT three months later again—this time without watching the relevant episode SMJ allowing us to map how the implicit preference change over time.

The experiment was conducted among MBA subjects at two reputed management training institutes in Delhi in India. MBA subjects provide an interesting subject pool for this experiment. The students are geared towards a private sector career where not only is there no caste-based reservation but representation of the lower castes is also small. The subject pool is also overwhelmingly upper caste and come from higher income strata. Unlike groups which are statedly biased and openly discriminate against lower castes, subjects in our sample represent that part of the population who most likely perceive themselves to be “neutral” and “unbiased” between castes but resist affirmative action mainly on grounds of efficiency.

Groups of students were randomly assigned to treatment or control. The instructions carefully explained the nature of the test. Since IAT required the test taker to associate different surnames with left and right categories, several examples of surnames and their caste affiliations were discussed. The experiment was done in two phases—in phase one (conducted in October, 2013 at International Management Institute), the treatment group had 29 subjects while the control had 30 subjects; in phase two (conducted in January, 2013 at Institute of Management Technology), the treatment and control groups had 18 and 17 subjects, respectively. Thus in all, the treatment and the control groups had 47 subjects each. However, out of the 29 subjects who had appeared in the treatment group phase one, 25 subjects came back to retake the test after 3 months (attrition rate was only 13% and the average rank of those who were unavailable was 2). Given this data and anecdotal investigations into the reasons for attrition, we believe that selection will not be an issue in the subsequent empirical analysis. We did not recall students who were in the control group since it was not clear what the interpretation of the longitudinal effect would be for a group who was not treated in the first place.

Subjects were recruited by word-of-mouth campaign and were informed that they would take part in a study on economic decision making. Though they had the choice of leaving the experiment at any point (but without getting paid), none of them did. They were paid Rs. 300 each for participating in the experiment for a session which lasted between 50 minutes and one hour. Subjects who took the IAT again three months later, were paid Rs. 200.

## Results

**Fig 3:**
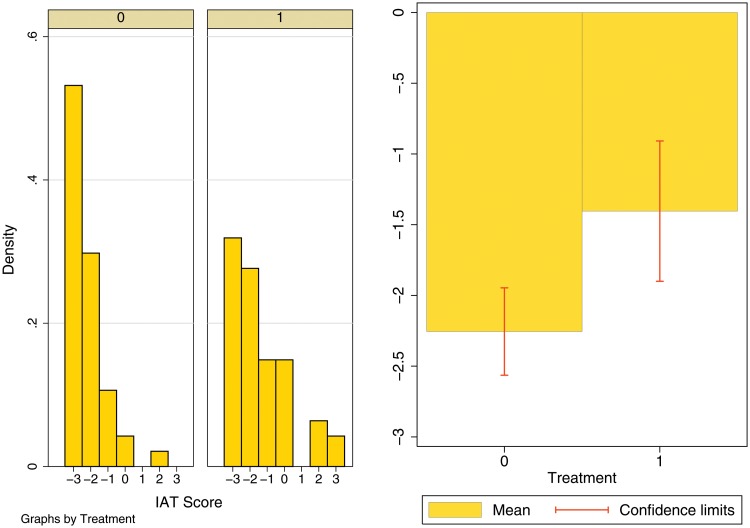
Distribution of IAT Level by Treatment status. Treatment 0 refers to the control group i.e. the group which watched Tom and Jerry. Treatment 1 refers to the treatment group i.e. the group which watched the Dignity for All episode of Satya Meva Jayate.

Fig 3 shows the distribution of the IAT levels by treatment status. Visual inspection reveals that the IAT level distribution for the treated group has more variance and is clearly right-translated compared to the distribution for the control group. We formally test the difference in the distributions by treatment status. We perform the Jonckheere-Terpstra test which tests the null hypothesis that the distribution of the response variable does not vary across treatment groups. For our sample, the null is strongly rejected (*p*-value = 0.007).

Descriptive statistics on the dependent variable and controls, separated by treatment status are shown in Table 1 below. The dependent variable is the IAT Level which ranges from -3 (strong automatic preference for Forward Caste compared to Scheduled Caste) to 3 (strong automatic preference for Scheduled Caste compared to Forward Caste). Both Treatment and Control groups show an implicit preference for the Forward Caste. However, the mean difference in IAT levels between Treated and Control is positive and highly significant, meaning that the Treated are more positively inclined toward the Scheduled Caste. The observed difference is not an institute specific feature—the average IAT levels in IMI are -2.3 and -1.2 while those in IMT are -2 and -1.6, for control and treatment groups, respectively. The magnitude of the difference corresponds to nearly one IAT level. Thus, while the Control group on average has a moderate automatic preference for Forward Caste compared to Scheduled Caste, the Treated group has a slight automatic preference for Forward Caste compared to Scheduled Caste.

**Table 1:** Descriptive Statistics.

	Control		Treatment		Difference
	Mean	S.D.	Mean	S.D.	
IAT level	-2.26	1.05	-1.4	1.69	0.851**
Share of Female	0.34	0.48	0.38	0.49	-0.04
Age	23.81	1.66	24.3	1.25	-0.49
Sec. exam level	86.93	6.08	87.06	5.68	-0.13
Watched show before	0.32	0.47	0.51	0.51	-0.19
Religiosity	2.53	0.88	2.6	0.92	-0.06
Reservation for Public Sector	0.27	0.45	0.4	0.5	0.13
Reservation for Private Sector	0.04	0.2	0.19	0.32	0.15**
N	47		47		

Note:

*** p<0.01,

** p<0.05,

* p<0.1 corresponding to mean difference using a t-test.

There are no significant differences according to treatment status in any of the control variables—i.e. share of female, age, secondary level exam level, religiosity (measured on an integer scale of 1 to 4 where 1 is Not at all religious and 4 is Highly religious) or whether subjects had watched the show before. In both groups, around two thirds of the subjects are male, the mean age is around 24 years and the score on their (national) secondary level exam is 87%, indicating that the subject pool is drawn from the higher end of the ability distribution. While 51% of the Treatment group had watched the show before, only 32% of the Control group had done so. The difference in the two shares is not, however, statistically significant. We can infer from these descriptive statistics that our subject pool, consisting of 1st and 2nd year business students drawn from business school in Delhi, is a homogeneous one. Note that we may be concerned that the Control group who was not exposed to the show may not have recalled watching it in the post-experiment survey. We cannot rule out that ‘Watched show before’ could be measured with error. This may potentially bias its effect downwards as well any variables correlated with it. The regression results are, however, robust to the addition of this variable, allaying any concerns about the presence of substantial measurement error.

### Regression Results

Results from simple OLS regressions treating the IAT level as a continuous variable are presented in Table 2 below. Control variables are added in successive specifications but improve the fit only marginally, which is to be expected based on the findings of the means analysis above. The basic result is that being exposed to the treatment (watching the TV show on caste injustices) increases the IAT level by around 0.87, i.e. nearly a full IAT level, which corresponds roughly to the difference in raw means. The only other significant estimate is on previous exam level and shows that the higher the secondary exam level the lower is the IAT level. It is worth noting though that the relation between IAT levels and secondary exam score is not strictly linear. This is evident from including a quadratic term of the exam score in the regression which shows that beyond 85% exam score caste bias starts to decrease again (not reported). Thus, the more high achieving students show a larger caste bias—a percentage point increase in secondary exam score leads to an increase of about 5% of an IAT level. The ordered nature of the dependent variable suggests an ordered categorical model (ordered probit) would be more appropriate than OLS. Model (6) estimates an ordered probit model and the main result remains unchanged. In a small sample such as this one, we are naturally concerned about the influence of outliers. The results of outlier tests based on the interquartile range showed no presence of serious outliers in the IAT level.

**Table 2:** Regression Results.

	(1)	(2)	(3)	(4)	(5)	(6)
Treatment	0.851***	0.860***	0.866***	0.900***	0.865***	0.732***
	(0.290)	(0.291)	(0.298)	(0.294)	(0.306)	(0.246)
Age			-0.0110	-0.0748	-0.0616	-0.0912
			(0.106)	(0.109)	(0.113)	(0.0896)
Sec. Exam Score				-0.0529*	-0.0511*	-0.0461**
				(0.0268)	(0.0272)	(0.0211)
Watched show before					0.157	0.162
					(0.309)	(0.239)
Religiosity					-0.0558	-0.0341
					(0.166)	(0.129)
Constant	-2.255***	-2.182***	-1.916	4.162	3.781	
	(0.205)	(0.230)	(2.562)	(3.981)	(4.127)	
Observations	94	94	94	94	94	94
R-squared	0.085	0.090	0.091	0.129	0.132	0.056

Note: Numbers below the coefficients represent the Standard errors.

*** p<0.01,

** p<0.05,

* p<0.1.

Outlier test showed 0.00% severe outliers. R-squared in (6) refers to pseudo-R-squared.

### Validation Exercise

Since the IAT test measures implicit or deep-seated bias, we may worry that simply being exposed to a one 1/2 hour TV show induces a potentially spurious or short-term change in the treated subjects’ preferences for the Scheduled Caste compared to the Forward Caste. We will explore this question in two ways: (1) We validate the findings above by comparing the differences in answers to two survey reported questions on whether the subject is in favor of public and private sector reservation for the lower castes. This also allows us to examine Prediction 1 from Section 2, namely a reduction in the discrimination leads to an increase in lower caste labor hiring (as proxied by job reservation opinion). (2) As mentioned earlier, we test the longer-run effect of the awareness program on implicit bias by asking treated subjects to take the IAT test 3 months after watching the TV show, but without watching the relevant episode SMJ again.

Regarding (1), unlike in the IAT which tests automatic associations, subjects have time to think about the questions on whether they are in favor of job reservation in the public and private sector. Thus their answers to these questions hence would reflect a more reasoned, conscious, and analytic belief.

**Fig 4:**
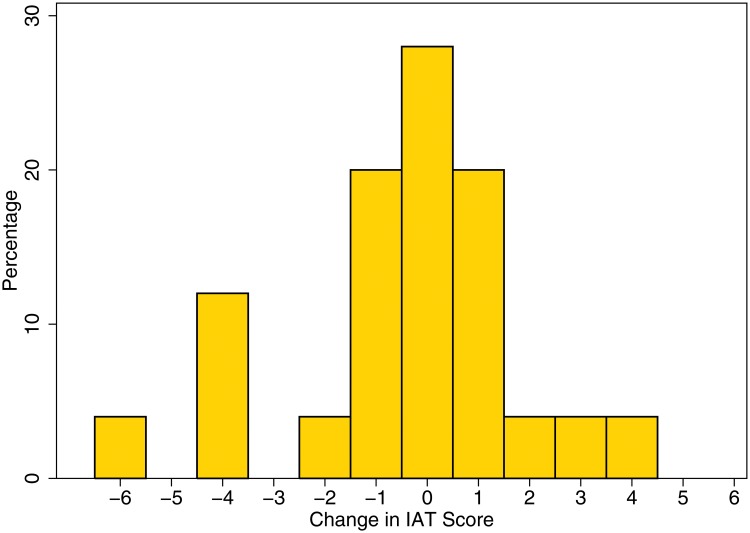
Distribution of the change in IAT level after three months.

40% of the subjects in the treatment group favored reservation in the public sector compared to 28% in the control group. A test of the difference showed that it was not statistically significant, Pearson Chi.sq(1) = 1.706 (Pr = 0.192). When it came to private sector reservation, 19% of the treated vs. 4% of the controlled were in favor. This difference was statistically significant at the 5% level, Pearson chi sq. (1) = 5.045 (Pr = 0.025). Since public sector reservation is already in place, the issue of introducing private sector reservation is possibly a more contentious one. In this case, we find that the treated group (who is otherwise identical to the control group) displaying a significantly stronger preference for reserving jobs in the private sector for the Scheduled Castes. Regarding (2), Fig 4 shows the distribution of the change in the IAT level after 3 months for the subset of treated individuals who took the re-test. 60% of the retake sample shows either no change or a positive change (more positive towards Scheduled Caste) in their IAT level. This breaks down to 28% showing no change in their IAT level compared to the last test, and 32% having a change of >1 IAT levels compared to the last test. 40% show a deterioration of their IAT level meaning an increase in implicitly held caste bias. Half this group, however, levels only 1 IAT level lower. We can conclude that a clear majority of our sample show no change or improve their IAT level 3 months later. Only 25 treated subjects participated in the 3-month follow-up even though they represent 86% of the first treated group. Whether these individuals participated because they have a more positive attitude towards the Scheduled Caste than other treated subjects in their group or because they responded more to the financial incentive cannot, unfortunately, be determined. There are, however, no significant differences in the compositional characteristics of the follow-up group compared to the treated group they were drawn from.

Finally, we also found that retakers’ views on public and private sector reservation showed persistence over time. 40% (25%) of retakers favored public (private) sector reservation, which matches almost exactly what we found 3 months earlier for the treatment group as a whole except that retakers were even more positive about private sector reservation (25% vs. 19%).

### Mechanisms

Our results quite convincingly show that the awareness promoting TV program reduces treated subjects’ implicit caste bias both in the short and longer-run. In this section, we explore some causal mechanisms. The reality show, Satya Meva Jayate (SMJ) was hosted by one of Bollywood’s most popular current film stars, Aamir Khan. It is possible that a celebrity effect is at play whereby subjects are strongly influenced to adopt the values and opinions endorsed by a celebrity. Celebrities are perceived to be more trustworthy and credible than non-celebrities (a halo effect) and may come across as possessing a more authentic connection to the behavior or product they are endorsing even though they do not possess any particular expertise in that area. For instance, individuals are far more likely to accept health advice from celebrities even though they may be ill-informed on these issues [25]. This may arise in part through celebrities giving a clearer signal by inducing greater recall of the product and thereby enabling individuals to differentiate between otherwise similar products [26]. Celebrities may also lead the herd in getting individuals to imitate their choices leading to an informational cascade [27]. In our set-up, subjects watched the TV program individually at their own computer workstations and were not allowed to interact or to discuss the contents with other subjects ruling out herd effects through informational cascade. Neither do we believe that a strong halo effect is present. If this were the case, subjects watching the show for the first time would be affected to a greater extent by this celebrity factor. As mentioned earlier, the control for having watched the show before was positive, but most likely due to low power, was insignificant in the regressions. Furthermore, estimating the IAT level regression on the two subsamples separately (watched before, did not watch before) produced very similar treatment effects—the estimate is 0.84 (Prob>|t| = 0.09) for the sample who had watched the show before and 0.79 (Prob>|t| = 0.04) for that who had not watched the test before. Formally conducting a Chow test of equality of the treatment effect and group effect across the two subsamples failed to reject the null F(2,90) = 0.24, Prob > F = 0.7905. The possibility that we are picking up an experimenter demand effect is minimal too since automatic preferences, as measured by IAT, cannot be altered consciously.

We believe, instead, that the treatment effect is a direct effect of the content of the program reducing taste-based discrimination against the lower castes in India. One of the central predictions of Becker’s theory of employer discrimination is that the relative hiring gap and the relative wage gap between majority and minority workers will be driven by the discriminatory preferences of the marginal employer who is just indifferent to hiring a majority or a minority worker, assuming firm sizes are all the same. This is because when the supply of minority workers is low, all minority workers will find employment with non-prejudiced employers who will pay them the same wage as majority workers. Where the wage gap arises is when the supply of minority workers exceeds the demand for them by non-prejudiced employers, forcing them to work (at lower wages) with prejudiced employers. The first prejudiced employer who is willing to hire a minority worker at a lower wage thus establishes the relative wage of minority and majority workers and hence the wage gap (Prediction 3). As it turns out, this is the not the most prejudiced employer but in fact the least prejudiced among the group of prejudiced employers.

Empirical support for this proposition has been found for the black-white wage gap in the U.S. [4] and for the ethic wage gap in Sweden [5]. [4] was the first to empirically apply Becker’s suggestion that the prejudice level of the marginal employer can be found at the *p*−th percentile of the majority employer prejudice distribution where *p* is the share of the workforce that consists of minority employees. Both papers access survey self-reported measures of prejudice and construct prejudice distributions on samples of individuals, not necessarily employers. [4], however, do separate analyses for the high-educated (at least a college education) in their sample, who according to them are more likely to be employers than the average person. Individuals in our sample, similarly, are soon-to-be graduates of a prestigious business school in Delhi and represent a group of potential future employers. We estimate a quantile regression of the treatment effect on the prejudice level (measured as -1 times IAT level and thus the expected signs in Table 3 are opposite to that of Table 2) to check whether the treatment effect we find arising in the experiment originates in the lower tail of the prejudice distribution. The results appear in Table 3 below.

**Table 3:** Quantile Regression of the prejudice level on treatment.

Variables	10th	25th	40th	50th	60th	75th	90th
treatment	-3***	-2**	-1	-1	-1	0	0
	(1.032)	(0.765)	(0.911)	(0.861)	(0.795)	(0.211)	(0.0860)
Constant	1	2***	2***	3***	3***	3***	3***
	(0.786)	(0.566)	(0.603)	(0.539)	(0.432)	(0.135)	(0.0549)
Observations	94	94	94	94	94	94	94

Note: Numbers below the coefficients represent the Standard errors.

*** p<0.01,

** p<0.05,

* p<0.1.

Each column shows the result from *p*-th quantile rgeression.

Here we can see that the treatment effect arises only at the left tail of the prejudice distribution of potential employers. For the treated group the prejudice is 3 and 2 levels lower for the control group and significant at the 10th and 25th percentile respectively but not significant elsewhere in the distribution including the median. It should be pointed out the sample is overwhelmingly Hindu upper caste, so the distribution approximately simulates the prejudice distribution of majority employers. There were 2 lower caste subjects and 1 Muslim subject—leaving them out does not change significance in mean difference in IAT ranks between treatments *p*−value = 0.01. The share of Scheduled Caste and Scheduled Tribe in the urban India’s population is 15.4% while that in rural India is 30% (Census 2011, Government of India.). The proportion of lower caste in the workforce is approximately similar and thus awareness programs are capable of reducing the prejudice of at least the marginal employer. Further, the result from Table 3 above, coupled with the earlier finding that the treatment induced a desire to increase private sector reservation, implies that employer caste bias against the lower castes in India is taste-based because the findings accord with the predictions of Becker’s employer discrimination model. Unlike the papers mentioned earlier, however, we are only able to test this on the employers’ hiring preference and not for wage inequality. As Prediction 1 in the theory section shows, this is an equivalent test of the theory of taste-based discrimination.

## Conclusion

We expose business school students at two prominent business schools in Delhi to either a TV program on caste injustices or an innocuous TV cartoon program. Following the viewings, we test subjects’ implicit caste biases via a caste IAT available through the Project Implicit website. Our results show that the treated group who watched the TV program showed significantly lower implicit bias and stated a higher preference for private sector job reservation. We also tested whether a longer-run treatment effect existed for a subset of the treated individuals and found that the lower bias levels either persisted or reduced even further 3 months after treatment for a majority of this group. That there is some evidence that the effect persists need further investigation in the future. Clearly, unlike priming, which is a subtle intervention with a short term effect, this intervention seems to have a deeper bite. It is not entirely surprising though, since the intervention had a clear, strong moral content and may have set the subjects thinking about why they fared in the IAT as they did. However, the deeper processes involved in the observed long term persistence of effects need further investigations. We also argued that the effect does not come from the fact that a celebrity actor hosted the program as the treatment effect was identical across the group who had watched the show before and the group who watched the show for the first time. We bring further evidence that the treatment shifted the prejudice distribution of our sample of highly-educated business school students (likely future employers) mainly at the lower tail. This fact, coupled with the additional finding that the treatment significantly increased a desire for private sector reservation, indicates that caste-based discrimination within this group is largely taste-based. It points to a potentially powerful role that awareness programs can play in reducing such bias. The results from our study can be generalized to apply to employers in urban areas. Reducing biased attitudes among employers would in turn lead to a reduction in caste discrimination in employment, which will have a large impact on economic productivity and efficiency. Since rapid growth is also taking place in suburban and rural areas and not just in cities, it will be useful to extend this analysis to target potential employers in these areas also. Television programs showing emancipated female characters have been found to bring about female empowerment in rural areas, so the potential for reducing caste bias and hence caste discrimination in these areas via awareness programs could be large as well.

## Instructions and Procedural Details

### Procedural Details

The recruitment process was done by word of mouth and subjects were told that they would be participating in an experimental study on psychology/economics. The experiment was conducted in a large lecture hall and subjects watched the shows on their individual computers. They also had access to the website where the instructions were laid out and links to the videos, tests and surveys were provided. Subjects were sufficiently spaced out from one another and other standard protocols of experiments were followed. This is an entirely between subject study i.e. subjects who took part in one treatment did not take part in another.

### Instructions for Satya Meva Jayate (Website link given below)

**Welcome!**

You are now taking part in an economic experiment. If you read the following instructions carefully, you can, depending on your decisions and the decisions of other participants, earn a considerable amount of money. It is prohibited to communicate with the other participants during the experiment. Should you have any questions please raise your hand and we will come to you. Please note down your identity number which you will require for payment and filling out your responses.

**Overview**

The study consists of an experiment and a survey.

**Earnings**

You will earn Rs. 200 for participating in this experiment.

**Details**

You will watch a video from youtube for the next half an hour. The video is part of the episode “Dignity for all” taken from Satya Meva Jayate (SMJ). Following that you will be asked to take a computer based simple test. You will receive a feedback from the test which you must write down on the sheet of paper which is provided to you. After that you will fill out a survey questionnaire. In the survey questionnaire you will be asked to state your result from the test. You must report the result and fill out the rest of the questions.

Please follow the following simple steps.

Step 1: Please click here to go to the following video links. We will watch video “Amir Speaks”, parts of 1 (2:30 to 12:00), 4 (1:00 to 11:00) and 5 (0:00 to 3:00).

Step 2: Please click here and take the test.

Click on Caste and then proceed. Please skip the survey by clicking OK at the bottom of the page.

Please write down the result of the test on the response sheet provided to you.

Step 3: Now please fill out one short survey. Survey for IAT click here.

### Instructions for Tom and Jerry (Website link given below)

**Welcome!**

You are now taking part in an economic experiment. If you read the following instructions carefully, you can, depending on your decisions and the decisions of other participants, earn a considerable amount of money. It is prohibited to communicate with the other participants during the experiment. Should you have any questions please raise your hand and we will come to you. Please note down your identity number which you will require for payment and filling out your responses.

**Overview**

The study consists of an experiment and a survey.

**Earnings**

You will earn Rs. 200 for participating in this experiment.

**Details**

You will watch a video from youtube for the next half an hour. The video is an episode of the classic Tom and Jerry. Following that you will be asked to take a computer based simple test. You will receive a feedback from the test which you must write down on the sheet of paper which is provided to you. After that you will fill out a survey questionnaire. In the survey questionnaire you will be asked to state your result from the test. You must report the result and fill out the rest of the questions.

Please follow the following simple steps.

Step 1: Please click here to go to the youtube video. You will now watch the Tom and Jerry video for the next 24 minutes.

Step 2: Please click here and take the test.

Click on Caste and then proceed. Please skip the survey by clicking OK at the bottom of the page.

Please write down the result of the test on the response sheet provided to you.

Step 3: Please fill out one short survey. Survey for IAT click here.
